# In Vitro Anti-Cholinesterase and Antioxidant Activity of Extracts of *Moringa oleifera* Plants from Rivers State, Niger Delta, Nigeria

**DOI:** 10.3390/medicines5030071

**Published:** 2018-07-05

**Authors:** Lucky Legbosi Nwidu, Ekramy Elmorsy, Jonah Sydney Aprioku, Iyeopu Siminialayi, Wayne Grant Carter

**Affiliations:** 1 Department of Experimental Pharmacology and Toxicology, Faculty of Pharmaceutical Sciences, University of Port Harcourt, Port Harcourt PMB 5323, Rivers State, Nigeria; sydaprio@yahoo.com; 2 Department of Forensic Medicine and Clinical Toxicology, Faculty of Medicine, Mansoura University, Mansoura 35516, Egypt; elkramy_elmorsy@yahoo.com; 3 Department of Pharmacology, Faculty of Basic Medical Sciences, University of Port Harcourt, Port Harcourt PMB 5323, Rivers State, Nigeria; siminialayi@yahoo.com; 4 School of Medicine, University of Nottingham, Royal Derby Hospital Centre, Derby DE22 3DT, UK; wayne.carter@nottingham.ac.uk

**Keywords:** *Moringa oleifera*, anti-cholinesterases, DPPH radical scavenging, antioxidant, oxidative stress, Alzheimer’s disease

## Abstract

This study evaluated *Moringa oleifera* extracts from two locations in Niger Delta for in vitro anti-cholinesterase and antioxidant activities. Methanolic, aqueous and ethanolic extracts of *Moringa oleifera* were evaluated for inhibition of acetylcholinesterase (AChE) activity, antioxidant properties, and total phenolic and flavonoid contents using standard procedures. *M. oleifera* extracts possessed significant and concentration dependent AChE inhibitory activity for methanolic, aqueous, and ethanolic extracts. For the most potent extracts, the percentage AChE inhibition/IC_50_ (µg/mL) values were *Moringa oleifera* root methanolic extracts (MORME): ~80%/0.00845; *Moringa oleifera* root ethanolic extract 1 (MOREE1): ~90%/0.0563; *Moringa oleifera* root ethanolic extract 2 (MOREE2): ~70%/0.00175; and *Moringa oleifera* bark ethanolic extract (MOBEE): ~70%/0.0173. The descending order of AChE inhibitory potency of plant parts were: root > bark > leaf > flowers > seed. All *M. oleifera* methanolic extracts at a concentration of 1000 µg/mL displayed significant (*p* < 0.05–0.001) DPPH radical scavenging activity, with values of ~20–50% of that of ascorbic acid. The total phenolic content and total flavonoid content (TPC/TFC) of MORME, *Moringa Oju* bark methanolic extract (MOBME), MOREE1, MOREE2 and Moringa leaf ethanolic leaf extract (MLEE) were (287/254), (212/113), (223/185), (203/343) and (201/102) mg gallic acid equivalents/g and quercetin equivalents/g, respectively. There was an inverse correlation between plant extract AChE inhibition and total phenolic (*p* < 0.0001) and total flavonoid contents (*p* < 0.0012). In summary, this study revealed 5 of 19 extracts of *M. oleifera* that have potent in vitro anti-cholinesterase and antioxidant activities.

## 1. Introduction

Alzheimer’s disease (AD) is the most prevalent neurodegenerative disease and is associated with progressive and irreversible loss of cognitive abilities, memory loss, cognitive impairment, emotional dysfunction, and ultimately death [1]. AD accounts for 50–70% of all cases of dementias [2,3], and for which dementia such as AD is a major cause of disability in the elderly. The epidemic scale of dementia poses one of the major challenges on global public health systems and associated financial burden with the social care needed. In 2015, an estimate of the number of people living with dementia was 46.8 million, with an associated economic burden of 818 billion US dollars, with numbers expected to grow year on year [3,4]. The financial burden, coupled to the social stigma associated with the loss of cognitive abilities and dependency on others, imposes considerable psychological distress in patients as well as their families [5].

AD is characterized by the formation of senile plaques, composed mainly of amyloid β (Aβ), and neurofibrillary tangles (NFTs), composed of tau protein, in the hippocampus and cerebral cortex of afflicted humans [6,7]. These protein aggregates (Aβ and tau proteins) provoke neuronal damage and synaptic dysfunction [8,9], hence the inhibition of their formation remains a potential therapeutic approach for the treatment of AD [10].

These abnormal protein accumulations also underpin neuro-inflammatory responses, neuronal toxicity, cell death, and cerebral atrophy [11]. The pathogenesis of AD is complex and multi-faceted and includes oxidative stress [12], inflammation [13], mitochondrial damage and/or dysfunction [14] and a cholinergic signaling deficit [15]. Attempts to preclude the cholinergic deficit have triggered the development of cholinomimetics and acetylcholinesterase inhibitors to maintain cholinergic transmission in the AD brain. Drugs such as tacrine, rivastigmine, donepezil, huperizine A and physostigmine (eserine) have been employed to treat patients with mild to moderate AD. However, these drugs only alleviate cholinergic symptoms, and were not designed to address other mechanistic pathways involved in AD disease progression. In fact, oxidative damage has been suggested to be a primary event in AD [16]. Indeed, protein, lipid, and DNA oxidation have been observed in brain tissues of AD Patients [17]. 

To preclude the risk associated with synthetic antioxidants, the utilization of natural antioxidants has been advocated [18,19]. Since natural antioxidants are an essential component of health and might prevent or delay cell damage [20], they have been exploited as potential leads for the development of novel AD drugs [21,22]. Furthermore, the neuroprotective effects of natural antioxidants and nootropics [23], such as *Ginkgo biloba* [24] and *Bacopa monnieri* [25] have attracted considerable attention in the management of AD [26].

*Moringa oleifera* belongs to the family Moringaceae. It is commonly known as horse radish tree or drumstick tree. It is a small-medium sized tree, 10–15 m high; widely cultivated in East and Southeast Asia, Polynesia, and the West Indies. It is also indigenous to North West India and many countries in Africa, South East Asia, Arabia, the Pacific, South America, and the Caribbean Islands. It is a widely cultivated tree, and considered a multi-purpose plant [27]. Although 12 varieties of Moringa species exist, it is likely that *M. oleifera* is the most widely known [27]. Almost all the plant parts: root, bark, gum, leaf, fruit (pods), flowers, seed and seed oil have been marketed as herbal therapies for various ailments in the Niger Delta of Nigeria. Diseases treated include inflammatory and infectious diseases, as well as cardiovascular, gastrointestinal, and hematological and hepatorenal disorders [28]. In Thailand, the tender pods, fruits and leaves of *M. oleifera* have been consumed as vegetables for over 100 years, while the hot water extract of the dried roots has been taken orally as a cardiotonic and a stimulant against fainting [29]. The potential therapeutic values against cancer, diabetes, rheumatoid arthritis, and other diseases have earned this plant the name of “wonder tree” in Thailand [30].

Extracts from *M. oleifera* display multiple pharmacological activities, including anti-inflammatory [31,32,33,34], antibacterial [35], antioxidant [36], anti-cancer [37], hepatoprotective [38,39] and neuroprotective [40,41,42] activities. The leaves and fruits also possess hypocholesterolaemic activity in Wistar rats [43] and rabbits [44]. 

A number of phytochemicals are present within, and obtained from, the Moringa plant, and these may contribute to its broad biological activity. For example, tannins, saponins, alkaloids, flavonoids, phenols and glycosides are resident within the leaves [45]; tannins, steroids, flavonoids, alkaloids, glycosides, quercetin and terpenoids within the flowers [46]; gallic acid, catechins, epicatechin, ferulic acid, vanillin, caffeic acid, protocatecuic acid, cinnamic acid, phytosterol, quercetin, glycosides and phenols within the seeds [47]; procyanidins, aurantiamide acetate, 3-dibenzylurea, quercetin glycoside, rhamnoglucoside quercetin and chlorogenic acid within the roots; and, procyanidin, sterols, triterpenoids, glycosides, tannins, alkaloids, *β*-sitosterol and octacosanoic acid from the stem bark [48]. Hence *M. oleiferia* has been extensively studied because of its enormous potentials as a source of nutraceuticals of medicinal value [49,50,51]. The plants from various regions of the world such as India [28], Thailand [30], Jamaica [52] and Pakistan [53] have been pharmacologically characterized, including the in vitro evaluation of antioxidant activity [54].

However, we have focused our attention to the *M. oleiferia* plant found in the Niger Delta of Nigeria, a region impacted by enormous exploration and exploitation of petroleum. Our research goal was to characterize the pharmacological activity of different parts of the *M. oleifera* plant, and from two different localities, and to focus upon its potential in vitro anti-cholinesterase and antioxidant activities. 

## 2. Materials and Method

### 2.1. Plant Materials Collection and Identification

*Moringa oleifera* parts (leaves, seeds, roots, flowers and bark) were collected in March 2015. The plant was authenticated by Mr. Okeke Chimezie, a botanist of the Department of Plant Science and Biotechnology, University of Port-Harcourt, Nigeria. Two garden specimens from two different locations in Rivers state, Nigeria: a coastland (No. 5, Abuloma Road, in Port Harcourt), and Hinterland (Okpaka’s Compound, Elele Alumini, Port-Harcourt), Nigeria were used for this study. A plant voucher number (UPH/P/60) was deposited in the University’s herbarium. The samples were air-dried for seven days and then powdered using an electrical blender and grinder, S-742 (Saisho, Nanjing, China).

### 2.2. Preparation of Methanolic, Ethanolic and Aqueous Extracts of Moringa oleifera

*Moringa oleifera* plants were collected and powdered, and samples weighed separately, before maceration in methanol or ethanol for 72 hours, as follows: leaf (300 g in 1500 mL of solvent), bark (250 g in 800 mL of solvent), root (250 g in 800 mL of solvent), seed (120 g in 300 mL of solvent), flower (120 g in 400 mL of solvent), respectively. Macerated samples were shaken 3 times daily to assist solvation. Solutions were then filtered using double-layered gauze. Filtrates were dried *in vacuo* at 40–50 °C on a water bath to obtain the methanolic or ethanolic dry extracts. Single sample extracts were weighed, and the percentage yield for each sample recorded.

For the aqueous extract, powdered samples of *Moringa oleifera* were weighed and extracted by a soxlet extraction method using distilled water. At the end of extraction, aqueous extract solution was dried *in vacuo* to obtain dry aqueous extracts. Single sample extracts were weighed, and the percentage yield for each sample recorded.

Collectively, we have prepared 19 extracts (8 methanolic, 3 aqueous, and 8 ethanolic). The higher number of methanolic and ethanolic extracts reflected the common usage of these solvents in ethnomedicine. 

### 2.3. Chemicals

Acetylthiocholine iodide (ATCI), ascorbic acid, bovine serum albumin (BSA), 2,2-Diphenyl-1-picrylhydrazyl (DPPH), 5,5-dithiobis (2-nitrobenzoic acid) (DTNB), Folin-Ciocalteau Reagent (FCR), gallic acid, physostigmine, and *β*-tocopherol were all purchased from Sigma Aldrich (Irvine, UK). 

### 2.4. Animals

To provide a source of mammalian acetylcholinesterase (AChE) enzyme, rat brain homogenates were used. Rats were male F344 strain, weight 200–230 g, as reported previously [55,56,57]. The use of these animals received approval from the University of Nottingham Local Ethical Review Committee (2008) (study reference CHE 10), with procedures performed in accordance with the Animals Scientific Procedures Act (UK) 1986.

### 2.5. Assay for Acetylcholinesterase Inhibitory Activity

AChE activity was measured within a 96-well microtitre plate based on the Ellman [58] method. Forty µL of plant extracts at concentrations of 200, 20, 2, 0.2 and 0.02 µg/mL were mixed with 50 μL of 3 mM DTNB, 50 µL of AChE (1 mg/mL, Sigma, C3389, Irvine, UK) or rat brain homogenate (prepared at 10% (*w*/*v*) according to references [55,56], and 35 μL of 50 mM Tris-HCl (pH 8.0) containing 0.1% BSA, and samples incubated for 5 min at 37 °C. The reaction was initiated by addition of 25 μL of 15 mM ATCI resulting in the production of a 5-thio-2-nitrobenzoate anion read at 412 nm every 5 s for 10 min using a Spectramax microplate reader (ThermoFisher, Stafford, UK). 

To establish suitable optical density changes and linearity of signal, a 1:10 dilution of rat brain homogenate (in 10 mM Tris-HCl pH 8.0) was used for AChE measurements. This rat brain positive control for AChE activity was inhibited in a dose-dependent manner by either eserine or organophosphorus pesticides [57].

### 2.6. Determination of 2,2-Diphenyl-1-picrylhydrazyl (DPPH) Radical Scavenging Effects

A DPPH radical scavenging assay was employed to determine, by a spectroscopic method, relative plant antioxidant ability. Anti-radical activities of plant extracts were estimated, according to the method of Nwidu et al. [57]. Stock solutions of extracts (5 mg/mL) were prepared and diluted to final concentrations of 200, 100, 50, 25, 12.5 and 6.25 µg/mL in ethanol. One hundred and 60 µL of 0.1 mM DPPH in ethanol solution was added to 20 µL of the extracts or standard, and then mixed with 20 µL of H_2_O. *β*-tocopherol (as a control solution) over the concentration range of 1.56, 0.78 0.39, 0.195, and 0.0975 mg/mL was assayed under similar conditions. The mixtures were incubated at 37 °C for 40 min in the dark. Sample absorbance was read at 517 nm, as described in Nwidu et al. [57]. 

### 2.7. Reducing Power Capacity Assessment

The reducing capacity of plant extracts were estimated based upon their ability to reduce ferric ions (Fe^3+^) to ferrous ions (Fe^2+^). The concentrations of the plant extracts ranged from 6.25 to 50 µg/mL. Four µL of 5 mg/mL of each plant extract was mixed with 400 µL of phosphate buffer (0.2 M dibasic sodium phosphate and 0.2 M monobasic sodium phosphate buffer, pH 7.4) and 250 µL of 1% potassium ferricyanide added, and then the mixture was incubated at 50 °C for 20 min. Then, 250 µL of 10% trichloroacetic acid was added, and the samples centrifuged at 3000 rpm for 10 min. One hundred µL of the supernatant was mixed with 100 µL of water followed by the addition of 20 µL of freshly prepared ferric chloride solution. Samples were then read at 700 nm, according to Nwidu et al. [57]. L-Ascorbic acid was employed as a positive control antioxidant.

### 2.8. Determination of Total Phenolic Content

Quantitation of total phenolics was determined spectrophotometrically at 760 nm, based on a colorimetric measurement (Folin-Ciocalteu Reagent (FCR) method) as described in a previous publication [57]. Plant extracts were assessed across the concentration range of 1–100 µg/mL. Twenty µL of plant extract was added to 90 µL of water, followed by addition of 30 µL of FCR and then the samples were shaken vigorously in a plate reader. Within eight minutes, 60 µL of 7.5% Na_2_CO_3_ solution was added, and the samples incubated at 40 °C on a shaking incubator, before reading at 760 nm in a spectrophotometer. Gallic acid over the concentration range of 0.1–0.5 mg/mL was processed in a similar fashion to provide a standard curve.

### 2.9. Determination of Total Flavonoid Content

Total flavonoid contents of the plant extracts was also determined according to the previously published method [57]. Quercetin was used as a reference compound. Twenty µL of plant extract (5 mg/mL) in ethanol was mixed with 200 μL of 10% aluminum chloride solution and 1 M potassium acetate. The mixture was incubated for 30 min at room temperature, and then read at 415 nm, according to Nwidu et al. [57]. 

### 2.10. Statistical Analysis

Results are expressed as means ± SD. The concentration of plant extract producing 50% inhibition (IC_50_) was calculated using non-linear regression analysis. A one-way ANOVA with Dunn’s multiple comparisons post-test was used to compare group data sets. A Spearman rank-order correlation coefficient was used to assess the relationship between total phenolic content, total flavonoid content, antioxidant content, and inhibition of AChE activity. Statistical analyses were performed using GraphPad Prism (Version 5.3) for Windows (GraphPad Software, Inc., San Diego, CA, USA, www.graphpad.com), with a *p* value of <0.05 considered significant.

## 3. Results

### 3.1. Moringa Oleifera Acetylcholinesterase Inhibitory Activity

**Figure 1:**
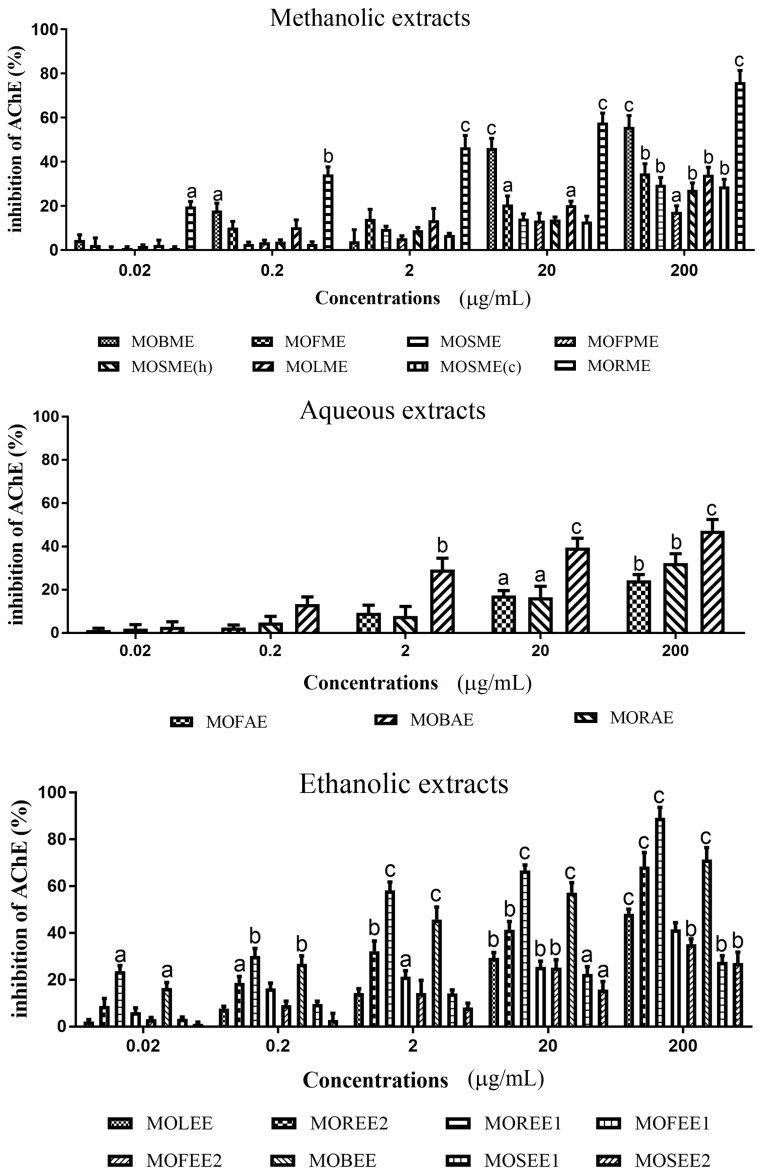
Acetylcholinesterase (AChE) inhibitory activity of plant extracts from *Moringa oleifera*. Plant extract inhibition of AChE was measured using a modified Ellman assay, with percentage inhibition of AChE calculated relative to eserine. The histograms presented are means ± SEM for at least three replicate assays at each extract concentration. a: *p* < 0.05. b: *p* < 0.01. c: *p* < 0.001.

*M. oleifera* extracts possessed significant and concentration dependent AChE inhibitory activity for methanolic, aqueous, and ethanolic extracts (Figure 1 and Table 1). For the most potent extracts, the percentage AChE inhibition/IC_50_ (µg/mL) values were *Moringa oleifera* root methanolic extracts (MORME): ~80%/0.00845; *Moringa oleifera* root ethanolic extract 1 (MOREE1): ~90%/0.0563; *Moringa oleifera* root ethanolic extract 2 (MOREE2): ~70%/0.00175; and, *Moringa oleifera* bark ethanolic extract (MOBEE): ~70%/0.0173. The descending order of AChE inhibitory potency of plant parts were: root > bark > leaf > flowers > seed. 

The descending order of AChE inhibitory potency for the *M. oleifera* methanolic extracts was MORME > MOSME (c) > MOBME > MOLME > MOFME > MOSME > MOSME (h) > MOFPME; for *M. oleifera* aqueous extracts was: MOBAE > MORAE > MOFAE; and for *M. oleifera* ethanolic extracts: MOREER2 > MOBEE > MOREE1 > MOLEE > MOSEE2 > MOFEE2 > MOFEE1 > MOSEE1 (refer to Table 1).

### 3.2. Moringa Oleifera DPPH Radical Scavenging Activity

**Figure 2:**
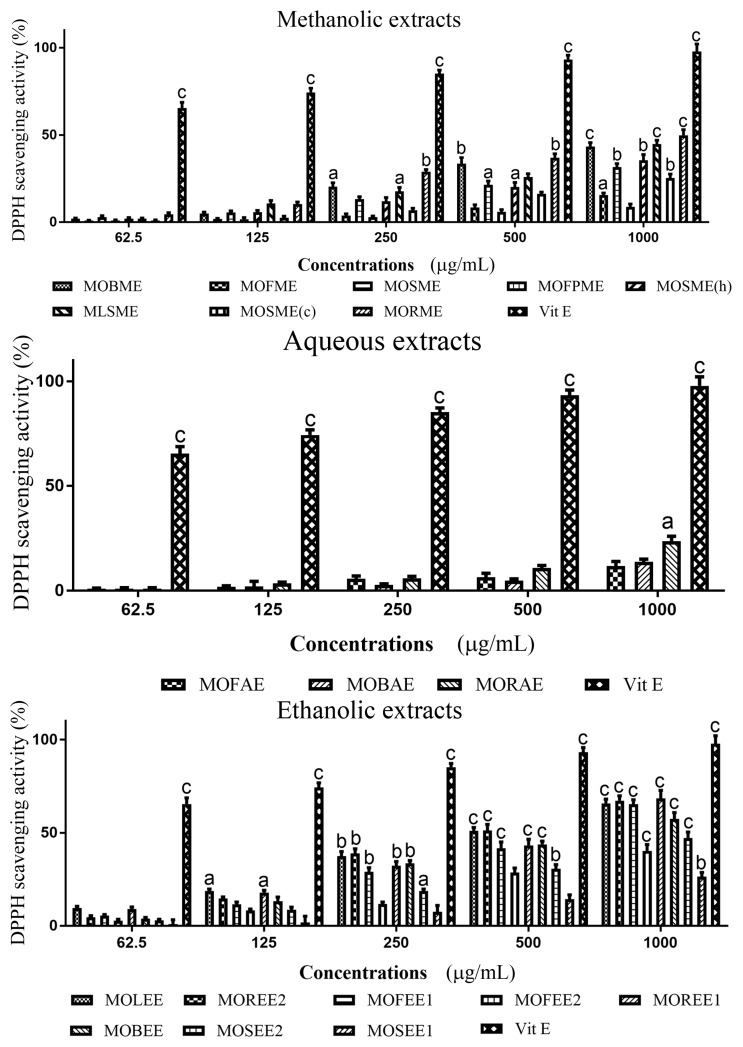
DPPH radical scavenging activity of plant extracts from *Moringa oleifera*. Plant radical scavenging activity was assessed using a DPPH radical, with results expressed as percentage inhibition. Vitamin E was used as a positive control. The histograms presented are means ± SEM for at least three replicate assays at each extract concentration. a: *p* < 0.05. b: *p* < 0.01. c: *p* < 0.001.

Methanolic, aqueous and ethanolic extracts of *M. oleifera* displayed DPPH radical scavenging activities in concentrations dependent manner, although for the aqueous fractions relatively low levels of radical scavenging was apparent (Figure 2 and Table 1). At a concentration of 1000 µg/mL, all *M. oleifera* methanolic extracts exhibited significant (*p* < 0.05–0.001) radical scavenging activity from ~20–50% of that of ascorbic acid (set at 100%) (Figure 2). The descending order of radical scavenging for the methanolic extracts was: MOLME > MOFPME > MOSME (h) > MOFME > MOSME > MOSME (c) > MOBME > MORME; for the aqueous extracts: MOBAE > MOFAE > MORAE; and ethanolic extracts: MOREE1 > MOSEE2 > MOREE2 > MOLEE > MOBEE > MOFEE1 = MOFEE2 > MOSEE1 (Table 1).

When considering the location of the plants, the MOREE1 from the coastland had a DPPH radical scavenging ability of ~70%/IC_50_ of 0.1176 × 10^−3^ mg/mL, whereas MOREE2 from the hinterland had a similar scavenging activity of ~70%, but with a higher IC_50_ of 0.4097 × 10^−3^ mg/mL.

### 3.3. Moringa Oleifera Reducing (Antioxidant) Capacity

**Figure 3:**
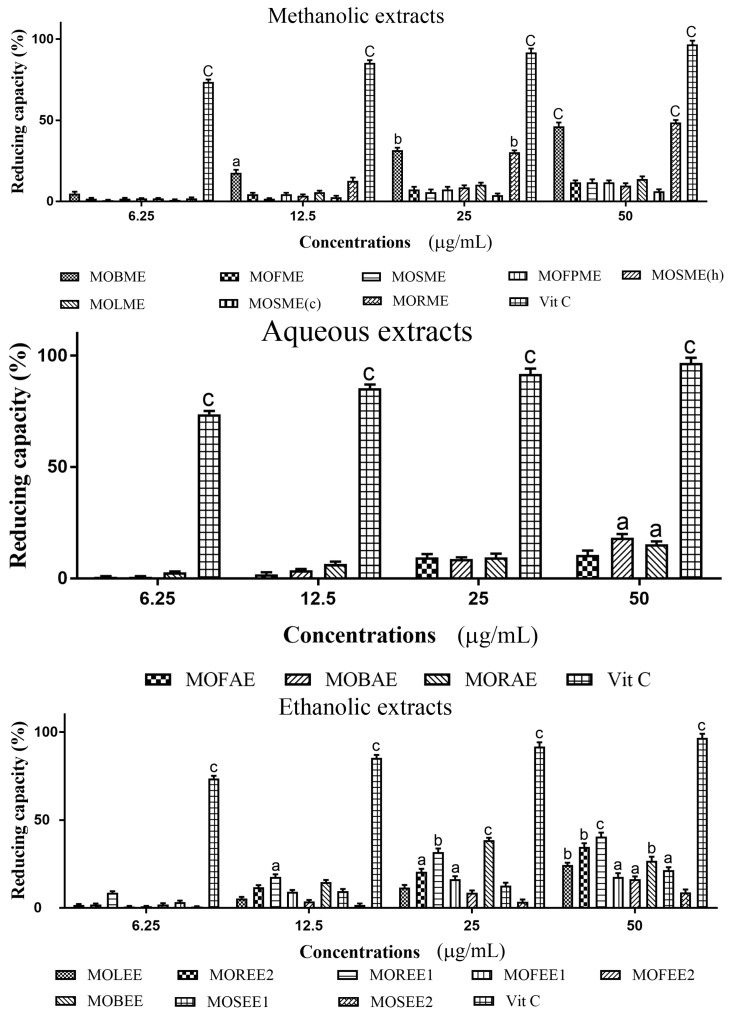
Reductive capacity of plant extracts from *Moringa oleifera*. Plant reducing power was quantified via the ability (as a percentage) to reduce ferric (Fe^3+^) to ferrous (Fe^2+^) iron. Ascorbic acid (vitamin C) was used as a positive control. The histograms presented are means ± SEM for at least three replicate assays at each extract concentration. a: *p* < 0.05. b: *p* < 0.01. c: *p* < 0.001.

Methanolic, aqueous and ethanolic extracts of *M. oleifera* displayed reducing (antioxidant) capacity in a concentrations dependent manner, although this was generally low relative to ascorbic acid (Figure 3). At an extract concentration of 50 µg/mL, of the methanolic extracts, only the MOBME and MORME displayed significant (*p* < 0.001) reducing capacity of ~50% compared to vitamin C set at 100%. Across the eight *M. oleifera* ethanolic extracts assayed, all except MOSEE2 exhibited significant (*p* < 0.05–0.001) reducing capacity. The highest antioxidant capacity for the ethanolic extracts was ~48% and ~45%, for MOREE1 and MOREE2, respectively. The order of descending reducing capacity for the *M. oleifera* methanolic extracts was: MORME > MOBME > MOLSME > MOFME = MOSME = MOFME > MOSME (h) > MOSME (c); and, for the aqueous extracts: MOBAE > MORAE > MOFAE; and ethanolic extracts: MOREE1 > MOREE2 > MOLEE > MOBEE > MOSEE1 > MOFEE1 > MOFEE2 > MOSEE2.

### 3.4. Moringa Oleifera Total Phenolic and Total Flavonoid Content

**Figure 4:**
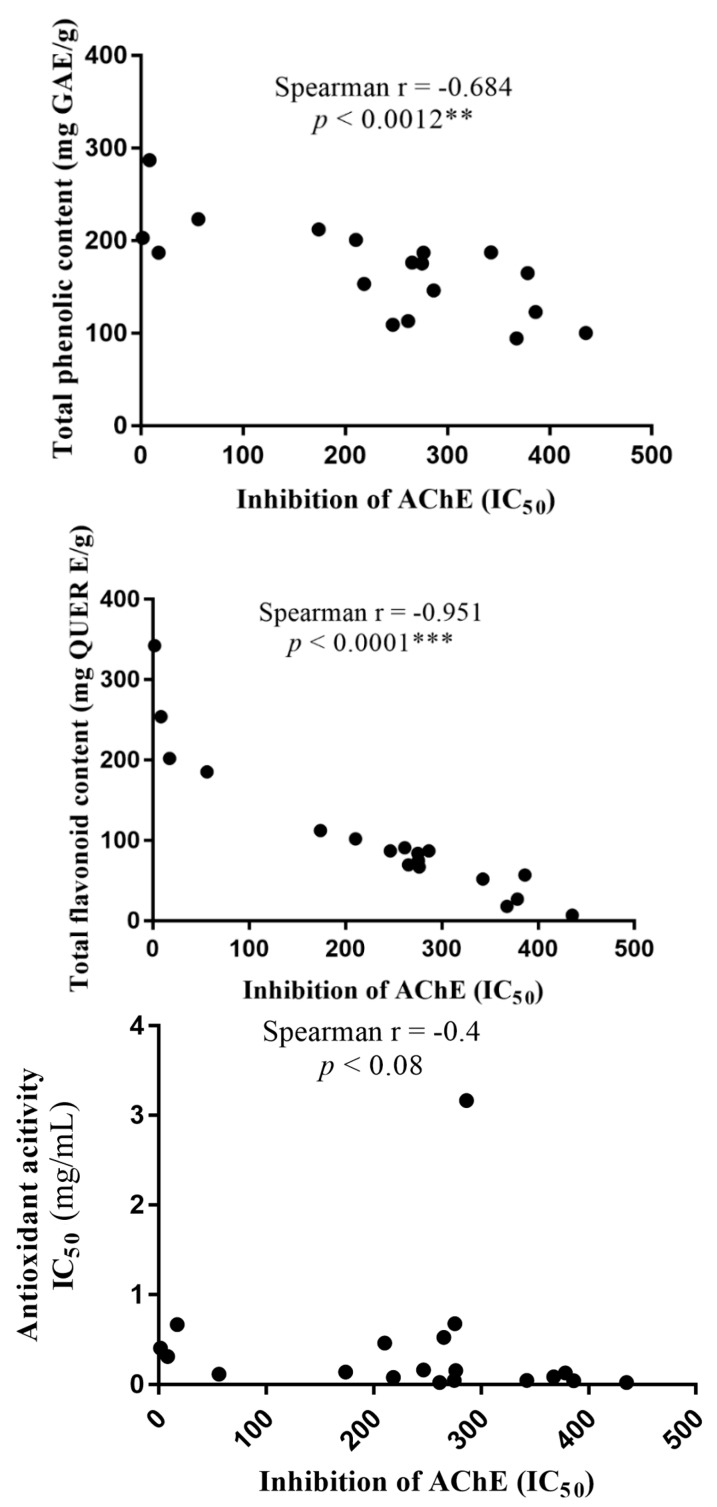
Spearman correlation of AChE inhibitory potency vs total phenolic content, total flavonoid content, and antioxidant potential for plant extracts from *Moringa oleifera*. Each dot represents one of the methanolic, aqueous, or ethanolic extracts. For significance, ** *p* < 0.01, *** *p* < 0.001.

Total phenolic content (TPC) and total flavonoid content (TFC) of the *M. oleifera* methanolic, aqueous, and ethanolic extracts were determined (Table 2). All fractions retained phenolic and flavonoid content, with the MORME extract displaying the highest levels of both compounds. Interestingly, there was a significant inverse correlation between total phenolic (*p* < 0.0001) and flavonoid content (*p* < 0.0012), and reduced potency of AChE inhibition (Figure 4). By comparison, there was no correlation between antioxidant capability and inhibition of AChE (Figure 4), or between antioxidant content and either TPC or TFC (results not included).

## 4. Discussion

*M. oleifera* neuroprotective effects have been reviewed [59,60,61,62] and experimentally demonstrated [40,41,63]. Various mechanisms, such as AChE inhibition, modification of monoamine levels, anti-amyloid aggregation, and antioxidant activities are strategies that have been employed for the amelioration of AD symptoms [60]. Of these, one of the major approaches has involved addressing the levels of acetylcholine in the brain that are depressed in AD using AChE inhibitors [64,65]. The cholinesterase inhibitors, eserine, tacrine, donepezil, rivastigamine, and galantamine, are known to have disparaging side effects that include disturbed sleep, diarrhea, nausea, headaches, and seizures [66,67]. As a result, there is intense scientific investigation to screen a plethora of plant extracts to discover more potent AChE inhibitors, hence this current evaluation of methanolic, aqueous, and ethanolic extracts of various plant parts (leaf, root, bark, flowers, etc.) of *M. oleifera* for anti-cholinesterase and antioxidant effects.

Many extracts and fractions of different plants, such as *Achyrocine tomentosa*, *Eupatorium viscidum*, *Ruprechtia apetala*, *Trichocline reptans*, *Zanthoxylum coco*, *Poncirus trifoliate*, *Treculia obovoidea*, *Angelica archangelica*, *Cassia obtisufolia*, *Salvia officinalis*, *Desmodium gangeticum*, and *Carpolobia lutea* have been assayed and reported to possess cholinesterase inhibitory activity [57,60,68]. However, this is not a universal property of plants *per se*, thus other plant extracts, for example, from *Sideroxylon obtusifolium*, *Erythrina velutina*, *Vitex agnus-castus* L., *Phoradendron piperoides*, *Chrysobalanus icaco*, *Bauhinia cheilantha*, and *Orbignya phalerata* do not exhibit any AChE inhibitory activity. Other plants, such as *Hyptis fruticosa* and *Maytenus rigida*, possess only low AChE inhibitory effects, whereas the plant investigated herein, *M. oleifera*, has ethanol leaf extracts with moderate AChE inhibitory activity; while, *Vitex agnus-castus* L. aqueous extract was an effective inhibitor of AChE [68].

A methanolic extract of *M. oleifera* with anti-cholinesterase effects has also been reported in vitro and in vivo in zebrafish (*Danio rerio*) [69], however, Moringa flower extract had no effect on gut AChE activity of insect larvae of *Aedes aegypti* [70]. Our explorative screening study revealed that *M. oleifera* methanolic, aqueous, and ethanolic extracts demonstrated considerable AChE inhibitory activity, that for some fractions was comparable to that induced with eserine. The MORME and MOBME from the methanolic extracts were the most potent AChE inhibitors with IC_50_ values of 0.00845 and 1.740 µg/mL, respectively. The MOBAE and MORAE from the *M. oleifera* aqueous extracts were the most potent, but had high IC_50_ values of 0.2764 and 0.215 µg/mL, respectively. Whereas the MOREE2, MOBEE and MOREE1 of the *M. oleifera* ethanolic extracts with IC_50_ values of 0.0173, 0.0563 and 0.00175 µg/mL, respectively, were the most potent ethanolic extracts.

Eserine (physostigmine) at a concentration of 0.02 µg/mL (~72 nM) was used as a positive control to completely inhibit electric eel or rat brain AChE, and for which relative inhibition of AChE by plant extracts was gauged. At this eserine concentration human brain AChE would likewise be inhibited ~100% (IC_50_ of ~14 nM) [57,71,72]. Hence, across the 19 screened *M. oleifera* extracts MOREE1, MORME, MOREE2 and MOBEE stand out as the majorly active AChE inhibitors with IC_50_ values of 0.00175, 0.00845, 0.0173 and 0.0563 µg/mL, respectively. These fractions show particular promise for further development and purification since in their partially purified form, they were more potent AChE inhibitors than eserine. 

To combat the multifaceted nature of neurodegenerative diseases such as AD, additional off-target actions such as radical scavenging and reducing (antioxidant) activities would be of benefit. Our results show that *M. oleifera* extracts, MOBME, MOLSME, MORME, MOLEE, MOFEE2, MOREE2 and MOREE1 significantly reduced DPPH radicals to about 50% of those of pure antioxidant Vitamin E. Superoxide anion radical (O^2−^) is a precursor to active free radicals that have the potential of reacting with biological macromolecules, and thereby inducing tissue damage [14]. In the assay undertaken, antioxidants react with DPPH, a purple colored stable free radical and convert it into a colorless *α-α*-diphenyl-*β*-picryl hydrazine. Plants with antioxidant properties, on interaction with DPPH, either transfer an electron or hydrogen atom to DPPH, thus neutralizing its free radical character, and changing the solution colour from purple to yellow. Our results that *M. oleifera* extracts displayed antioxidant properties was also validated by the ability to reduce ferric to ferrous iron. Mild, but useful reducing (antioxidant) capacity was apparent, in keeping with other studies that have quantified *M. oleifera* antioxidant capability [52,53,54].

In addition to establishing the potent AChE inhibitory activity and antioxidant properties of *M. oleifera* we quantified the total phenolic and total flavonoid contents. For the MORME extracts, a relatively high phenolic and flavonoid content was detected. *M. oleifera* extracts that demonstrated potent AChE inhibitory activity, also displayed significant antioxidant activities and contained a relatively high content of polyphenols and flavonoids. The natural antioxidants that are present in plants, such as *M. oleifera*, may inhibit or prevent the deleterious consequences of oxidative stress, and this could relate to certain phenolic and flavonoid contents [46]. For example, polyphenols inhibit lipid peroxidation by acting as chain-breaking peroxyl-radical scavengers [73]. For *M. oleifera*, certain terpenoids, steroids, and phenolic compounds such as tannins, coumarins and flavonoids could provide the proficient antioxidant properties [74,75].

Interestingly, the AChE IC_50_ inhibitory concentrations for *M. oleifera* extracts were significantly inversely correlated to total phenolic and total flavonoid contents (Figure 4). This suggests that the agent(s) responsible for the AChE inhibitory activity contain phenolic and flavonoid compounds. However, there was not a correlation between AChE inhibitory activity and antioxidant activity, or between antioxidant activity and TPC or TFC. Polyphenols may certainly possess antioxidant properties [76,77], but the antioxidant activities of flavonoids are variable, and may, for example, reflect the presence or absence of a catechol B-ring [78]. Hence, the discordance between TPC/TFC and antioxidant activity may simply reflect the specific phenolic or flavonoid compound components of *M. oleifera* that were retained while using our solvent systems. We employed methanol, ethanol, and water for extract dissolution based upon their extensive use as solvents in ethnomedicine [79,80], as they are relatively inexpensive, suitable for dissolution of both polar and non-polar compounds, and can be evaporated with ease facilitating extract concentration.

Collectively, the extensive armamentarium of phytochemicals of *M. oleifera* [45,46,47] likely contribute to the potent anti-cholinesterase and antioxidant effects we describe in this study. However, a limitation of our results is that the data only reflects an in vitro study. To date, preliminary in vivo findings have shown that a *M. oleifera* hydroalcohol leaf extract was capable of mitigating memory impairment in rats [40]. Yet follow-up studies that assess the ability of other *M. oleifera* extracts or purified compounds to ameliorate symptoms of cholinergic deficits in animal models of diseases such as AD, Parkinson’s disease, or myasthenia gravis are still required. Nevertheless, the impetus for in vivo studies should be based upon satisfactory in vitro data of potent anti-cholinesterase activity, and our study certainly suggests that *M. oleifera* reaches this criterion. 

## Figures and Tables

**Table 1:** Percentage yield, AChE inhibitory, and 2,2-Diphenyl-1-picrylhydrazyl (DPPH) radical scavenging potency of *Moringa oleifera* methanolic, aqueous and ethanolic extracts.

*Moringa oleifera* Extracts	Yield (%)	IC_50_ Concentrations (mg/mL)	
		AChE	DPPH Radical Scavenging (×10^−3^)
Methanolic			
MOBME	2.67	0.1740	0.1419
MOFME	8.88	0.2750	0.04767
MOSME	3.44	0.3425	0.04902
MOFPME	14.71	0.4335	0.02579
MOSME (h)	6.83	0.3863	0.04561
MOLME	4.78	0.2615	0.02517
MOSME (c)	3.68	0.08723	0.08723
MORME	9.84	0.00845	0.3148
Aqueous			
MOFAE	39.1	0.3784	0.1313
MOBAE	26.2	0.2185	0.08298
MORAE	14.3	0.2764	0.1574
Ethanolic			
MOLEE	5.3	0.2105	0.4638
MOREE2	1.1	0.00175	0.4097
MOREE1	6.3	0.0563	0.1176
MOFEE1	14.4	0.2756	0.6819
MOFEE2	3.1	0.2654	0.6819
MOBEE	10.2	0.0173	0.6709
MOSEE1	7.1	0.2864	3.168
MOSEE2	10.3	0.2464	0.1653

Methanolic extracts: MOBME, *Moringa oleifera* bark methanolic extract; MOFME, *Moringa oleifera* flower methanolic extract; MOSME, *Moringa oleifera* stembark methanolic extract; MOFPME, *Moringa oleifera* food powder methanolic extract; MOSME (h), *Moringa oleifera* stalk methanolic extract (hot); MOLME, *Moringa oleifera* leaf methanolic extract; MOSME (c), *Moringa oleifera* stalk methanolic extract (cold); MORME, *Moringa oleifera* root methanolic extract. Aqueous extracts: MORAE, *Moringa oleifera* root aqueous extract; MOFAE, *Moringa oleifera* flower aqueous extract; MOBAE, *Moringa oleifera* bark aqueous extract. Ethanolic extracts: MOLEE, *Moringa oleifera* leaf ethanolic extract; MOREE1, *Moringa oleifera* root ethanolic extract 1; MOREE2, *Moringa oleifera* root ethanolic extract 2, MOFEE1, *Moringa oleifera* flower ethanolic extract 1; MOFEE2, *Moringa oleifera* flower ethanolic extract 2; MOBEE, *Moringa oleifera* bark ethanolic extract; MOSEE1, *Moringa oleifera* seed ethanolic extract 1; MOSEE2, *Moringa oleifera* seed ethanolic extract 2. Extracts denoted 1 are *Moringa oleifera* plants from lowland, and extracts denoted 2 are *Moringa oleifera* plants from hinterland.

**Table 2:** Total phenolic and flavonoid content of Moringer oleifera methanolic, aqueous, and ethanolic extracts.

*Moringa oleifera* Extracts	Total Phenolic Content (mg GAE/g)	Total Flavonoid Content (mg QUER E/g)
Methanolic		
MOBME	212.3 ± 2.30	112.5 ± 2.40
MOFME	175.6 ± 0.09	84.3 ± 2.30
MOSME	187.4 ± 2.00	52.0 ± 0.60
MOFPME	100.4 ± 0.08	7.3 ± 1.90
MOSME (h)	123.2 ± 1.10	57.0 ± 3.30
MOLME	113.3 ± 1.90	91.2 ± 0.90
MOSME (c)	94.5 ± 0.90	18.0 ± 0.09
MORME	287.1 ± 0.00	254.3 ± 2.30
Aqueous		
MOFAE	165.2 ± 0.80	27.0 ± 3.00
MOBAE	153.3 ± 0.08	87.2 ± 3.60
MORAE	187.0 ± 1.90	67.2 ± 2.00
Ethanolic		
MOLEE	201.0 ± 2.30	102.2 ± 1.50
MOREE2	203.2 ± 0.02	342.5 ± 1.70
MOREE1	223.2 ± 1.01	185.4 ± 2.70
MOFEE1	186.3 ± 2.00	75.0 ± 0.30
MOFEE2	176.3 ± 0.30	69.7 ± 1.70
MOBEE	187.2 ± 2.00	202.3 ± 3.10
MOSEE1	146.3 ± 0.20	95.3 ± 2.5
MOSEE2	109.2 ± 0.80	87.2 ± 3.60

Extracts denoted 1 are *Moringa oleifera* plants from lowland, and extracts denoted 2 are *Moringa oleifera* plants from hinterland.
